# Unfluctuating diastolic pressure, stiff arteries – the unseen culprits behind the curtain of cognitive decline: An observational study

**DOI:** 10.1097/MD.0000000000044190

**Published:** 2025-09-05

**Authors:** Wen Zhong, Lingling Wang, Yiwen Xu, Mengqian Liu, Likai Jin, Yi Jiang, Xiaozhu Shen

**Affiliations:** a Department of Geriatrics, Lianyungang Hospital Affiliated to Jiangsu University, Lianyungang, China; b Department of Geriatrics, The Second Peoples Hospital of Lianyungang, Lianyungang, China; c Department of Infectious Disease, The First Affiliated Hospital of Ningbo University, Ningbo, China; d Department of Stomatology, Chikaijia Clinic of Lianyungang, Lianyungang, China; e Department of Geriatrics, Lianyungang Hospital Affiliated to Bengbu Medical College, Lianyungang, China.

**Keywords:** arteriosclerosis index, cognitive impairment, diastolic blood pressure variability

## Abstract

Cerebral small-vessel disease (CSVD) is an important risk factor for cognitive impairment, which is a pressing health issue for the aging population worldwide. The complex relationship between vascular factors, such as blood pressure variability (BPV) and arteriosclerosis index (AI), and cognitive dysfunction in patients with CSVD is a hot research topic, and research in this area will help prevent and treat cognitive dysfunction in CSVD. This study aims to investigate the effects of diastolic BPV (DBPV) and AI on cognitive function in patients with CSVD. A total of 383 CSVD patients admitted to the Second People’s Hospital of Lianyungang City from May 2018 to June 2022 were included in this study. Patients with CSVD were divided into 4 groups based on the Mini-Mental State Examination (MMSE) to compare the differences between these groups. AI = (blood total cholesterol – high-density lipoprotein cholesterol [HDL-C]) ÷ HDL-C; DBPV = standard deviation of 24-hour DBP (SD of 24h-DBP). A logistic regression model was constructed to screen out the risk factors for cognitive dysfunction in patients with CSVD, and the model was evaluated using the receiver operating characteristic curve. Patients with different degrees of cognitive dysfunction revealed differences in 24h mean diastolic blood pressure (DBP), DBPV, daytime DBP, nocturnal systolic blood pressure and nocturnal drop in systolic blood pressure and DBP between the groups, and the mean DBP standard deviation of the groups with mild to moderate cognitive dysfunction was lower than that of the group with normal cognitive function (*P* < .05).The mean DBP of the groups with moderate cognitive dysfunction was lower than that of the group with normal cognitive function (*P* < .05). DBPV had a negative correlation with the AI (*r* = −0.56, *P* < .001), and a positive correlation with the MMSE score (*R* = 0.18, *P* = .024). AI was negatively correlated with MMSE score (*r* = −0.26, *P* < .001). Multivariate logistic regression analysis revealed that when DBPV was 10.58 to 13.60 mm Hg as a protective factor for vascular cognitive dysfunction, the AI was a risk factor for vascular cognitive dysfunction. DBPV and AI are strongly associated with CSVD-based cognitive dysfunction.

## 1. Introduction

Cerebral small-vessel disease (CSVD) is a group of cerebrovascular lesions affecting microarterioles and small arterioles,^[1]^ and it is one of the most important causes of cognitive dysfunction and dementia in the elderly.^[2]^ Globally, the prevalence and mortality of CSVD are increasing year by year, imposing a huge burden on individual and social health.^[3]^

Cognitive dysfunction due to CSVD is inextricably linked to the health of the vascular system.^[4,5]^ The 2 key factors affecting vascular health are arterial blood pressure and the variability of the vessel wall structure. Arterial blood pressure plays a fundamental role in the mechanical stress exerted on the blood vessels, influencing their elasticity and overall function. On the other hand, the variability of the vessel wall, including factors like thickness, stiffness, and endothelial function, can reflect the presence of pathological changes that predispose to conditions such as atherosclerosis, hypertension, and CSVD. Together, these factors interact to determine the long-term health and integrity of the vascular system, influencing the risk of cardiovascular events and the development of vascular-related diseases.^[4]^ Blood pressure variability (BPV) refers to the degree to which blood pressure fluctuates within a given time frame, and is the result of a complex interaction between external environmental and behavioral stimuli, intrinsic cardiovascular regulatory mechanisms, and humoral and rheological factors.^[6,7]^ Previous studies have shown a clear association between BPV and CSVD.^[8–10]^ BPV is strongly associated with the risk of developing CSVD and its severity, and systolic BPV (SBPV) has been shown to be positively associated with the incidence of cerebral white matter lesions, stroke, and cognitive decline.^[4,11]^ SBPV leads to unstable cerebral perfusion, which may result in chronic underperfusion or intermittent overperfusion, both of which can impair brain microstructure and function over time.^[12]^ This instability exacerbates the effects of CSVD, a key pathological substrate for vascular cognitive dysfunction, leading to lacunar infarcts, microhemorrhages, and diffuse white matter lesions, all of which are associated with cognitive impairment.^[13]^

However, the above studies have focused on the effect of SBPV on CSVD, whereas the significant effect of diastolic BPV (DBPV) on CSVD is rarely reported. The interplay between DBPV and cognitive functions is multifaceted at the age where diastolic pressure is starting to decline. This complexity in variability may stem from the process of vascular aging, which is influenced by unique, individual factors not necessarily aligned with chronological age.^[14]^ After adjusting for multiple comparisons, it was found that a larger early and late-phase DBPV is associated with declines in attention tasks and psychomotor tasks, as well as a greater volume of white matter hyperintensities on imaging.^[15]^ It is generally accepted that DBPV fluctuations decrease with age, influenced by vascular aging.^[16]^ In this study, we aimed to investigate the relationship between DBPV and cognitive function, given that DBPV reflects fluctuations in DBP, which can be an indicator of vascular health. These fluctuations may be associated with impaired cerebral perfusion and may influence cognitive outcomes, particularly in individuals with CSVD. In this context, the arteriosclerosis index (AI) was introduced to assess the elasticity and stiffness of the vascular wall, as these factors reflect the degree of vascular aging and arteriosclerosis, both of which are critical in the pathophysiology of CSVD. By considering both DBPV and the AI, we aim to explore how vascular changes contribute to cognitive decline in CSVD patients, providing a deeper understanding of the interplay between BPV and cognitive function in vascular aging.^[17,18]^ Arteriosclerosis leads to endothelial rupture and vascular inflammatory response, which further affects the function and structure of the small blood vessels in the brain and accelerates the development of cognitive dysfunction.^[19]^

Based on the understanding of the interactions between DBPV and CSVD,the aim of this study was to investigate the relationship between DBPV and AI and cognitive dysfunction associated with CSVD, and to provide a scientific basis for the development of effective preventive and therapeutic interventions.

## 2. Methods

### 2.1. Research population

383 patients with CSVD admitted to the Department of Geriatrics of the Second People’s Hospital of Lianyungang City from May 2018 to June 2022 were selected. The diagnosis of CSVD was made according to the CSVD diagnostic criteria of the 2021 Expert Consensus on the Diagnosis and Treatment of CSVD, with cerebral magnetic resonance imaging revealing the presence of at least 1 type of white matter hyperintensity, enlarged perivascular space, lacunar infarct, subcortical infarct, and cerebral microbleed. Exclusion criteria: presence of other diseases diagnosed as cognitive dysfunction (Alzheimer disease, Parkinson disease, stroke, dementia, etc have been definitively diagnosed); those with a combination of severe infections or severe systemic diseases (cardiorespiratory insufficiency, hepatic and renal insufficiency, immune disorders, hematological disorders, etc); inability to complete the mini-mental state examination (MMSE) scores; clinical data were missing or incomplete (>30% missing data).

Written informed consent or informed consent from their legal guardians was obtained from all patients enrolled in this study, and the study received ethical approval from the Ethics Committee of the Second People’s Hospital of Lianyungang (NO. 2020021).

### 2.2. Data collection

#### 2.2.1. General information collection

General information about the patients was collected, including demographic information, MMSE scores, history of hypertension, history of diabetes, history of heart disease, history of stroke or transient ischemic attack, history of smoking, history of alcohol consumption, and history of medication.

#### 2.2.2. Laboratory examinations

All subjects fasted for more than 8 hours. Venous blood was drawn the following morning and fasting blood glucose, total cholesterol (TC), triglycerides (TG), low-density lipoprotein cholesterol (LDL-C), high-density lipoprotein cholesterol (HDL-C), and lipoproteins were measured. AI = [TC—HDL-C] ÷ HDL-C.

#### 2.2.3. Cognitive assessment

The enrolled patients were assessed for cognitive function within 48 hours after the diagnosis of CSVD, and we chose the MMSE as a screening tool to assess the cognitive function of the patients.According to the MMSE, the CSVD patients were categorized into cognitive dysfunction group (MMSE < 27 points) and normal cognitive function group (MMSE ≥ 27 points) according to the education level of the patients. Meanwhile, in order to better compare the relationship between vasodilatability and AI and the severity of cognitive dysfunction, the patients were divided into 4 groups according to the MMSE scores: cognitively normal group (27–30 points); mild cognitive dysfunction group (21–26 points); moderate cognitive dysfunction group (10–20 points); and severe cognitive dysfunction group (0–9 points). The general clinical data and biochemical indicators of these 4 groups of patients were then compared.

#### 2.2.4. Ambulatory blood pressure measurement

All subjects underwent 24-hour ambulatory blood pressure monitoring using a portable noninvasive ambulatory blood pressure monitor within 48 hours after admission to the hospital, and hypertensive patients continued to take their daily antihypertensive medication during the monitoring period. The cuff of the blood pressure monitor was placed on the upper arm of the patient’s nondominant or nonparaplegic side, and the blood pressure was set to be measured every 30 minutes during the daytime and every 1 hour during the nighttime, and the time was set to be 7:00 to 21:00 during the daytime and 21:00 to 7:00 during the nighttime, and the number of times of monitoring of blood pressure in the 24-hour period was >80% of the number of times that should be obtained as the valid data. Patients were required to have normal daily activities during the day and adequate rest and sleep at night. The 24-hour mean SBP (24h-SBP), 24-hour mean DBP (24h-DBP), daytime mean SBP (dmSBP), daytime mean DBP (dmDBP), nocturnal mean SBP (nmSBP), nocturnal mean DBP (nmDBP) were recorded, and the standard deviation (SD) of 24-hour systolic and DBP was calculated. Afterward, the ratio of nocturnal systolic and DBP reduction was calculated separately, with nocturnal SBP reduction rate = (dmSBP-nmSBP) ÷ dmSBP × 100% and nocturnal DBP reduction rate == (dmDBP-nmDBP) ÷ dmDBP × 100%. We used the SD of 24-hour DBP (SD of 24h-DBP) as a proxy for BPV by grouping them into quartiles (Quartile 1, <8.80 mm Hg; Quartile 2, 8.80 to 10.57 mm Hg; Quartile 3, 10.58 to 13.60 mm Hg; Quartile 4, >13.60 mm Hg).

### 2.3. Statistical analysis

Data were tested for normality using the 1 sample Kolmogorov-Smirnov test. Continuous variables that conformed to normal distribution were tested using the independent samples *t*-test and expressed as mean ± SD. Non-normal variables was assessed for between-group differences using the Kruskal-Wallis method and expressed as median and quartiles. Categorical data were expressed as frequencies and proportions using the chi-square test or Fisher exact test. Correlation analysis of participants’ clinical data was performed by Spearman correlation. Restricted cubic spline curves (RCS) were used to assess the dose relationship between variables and severity of cognitive function. A multifactorial logistic regression model was used to analyze the relationship between DBP, AI, and cognitive dysfunction, and the model was evaluated using the receiver operating characteristic (ROC) Curve. *P *< .05 was considered statistically significant. All statistical analyses were performed using R (version 4.2.3).

## 3. Results

### 3.1. Patients characteristics

A total of 383 patients with CSVD were included in this study, and the differences between groups with different degrees of cognitive dysfunction are shown in Table 1, including 159 patients in the group with normal cognitive function, 129 patients in the group with mild cognitive dysfunction, 72 patients in the group with moderate cognitive dysfunction, and 23 patients in the group with severe cognitive dysfunction. Patients with more severe cognitive dysfunction were relatively older and had relatively lower MMSE scores compared to the normal cognitive function group. Patients in the mild cognitive impairment group were more likely to have a history of stroke or transient ischemic attack. There were no differences in gender, smoking history, history of hypertension, history of diabetes, or history of cardiac disease between the groups. The use of antiplatelet drugs, antihypertensive drugs, anticoagulants, lipid-lowering drugs, and hypoglycemic drugs was similar among the 4 groups. In terms of laboratory indicators, the AI was higher in the moderate cognitive dysfunction group than in the other groups, and there was no difference between the groups in any of the other laboratory indicators. Comparison of blood pressure monitoring indexes in patients with different degrees of cognitive dysfunction found no difference between the 4 groups of 24h-SBP, SD of 24-hour mean SBP (SD of 24h-SBP), dmSBP and nmDBP, while there was a difference between the groups of 24h-DBP, SD of 24h-DBP, dmDBP, nmSBP, as well as SBP and DBP nocturnal drop rate (*P *< .05).

**Table 1:** Baseline characteristics according to varying degrees of cognitive dysfunction.

Characteristics	Total (n = 383)	Normal cognition (n = 159)	Mild cognitive impairment (n = 129)	Moderate cognitive impairment (n = 72)	Severe cognitive impairment (n = 23)	*P*-value
Age (years)	74.00 (65.00–83.00)	67.00 (59.00–74.00)	78.00 (70.00–84.00)	82.50 (68.75–88.25)	87.00 (82.00–91.00)	<.001*
Gender, male, n (%)	171 (44.65)	78 (49.06)	49 (37.98)	32 (44.44)	12 (52.17)	.251
Smoking, n (%)	68 (17.75)	34 (21.38)	17 (13.18)	16 (22.22)	1 (4.35)	.069
Drinking, n (%)	51 (13.32)	31 (19.50)	13 (10.08)	6 (8.33)	1 (4.35)	.022**
MMSE score	25.00 (21.00–28.00)	28.00 (28.00–29.00)	24.00 (23.00–25.00)	15.00 (11.00–18.25)	3.00 (0.00–6.00)	<.001*
Past medical history						
Hypertension, n (%)	306 (79.9)	132 (83.02)	95 (73.64)	61 (84.72)	18 (78.26)	.159
Diabetes, n (%)	92 (24.02)	42 (26.42)	30 (23.26)	18 (25.00)	2 (8.70)	.316
Stroke or TIA, n (%)	137 (35.77)	40 (25.16)	50 (38.76)	35 (48.61)	12 (52.17)	.001***
Cardiological history, n (%)	130 (33.94)	44 (27.67)	46 (35.66)	30 (41.67)	10 (43.48)	.121
Drug treatment history						
Antiplatelet drugs, n (%)	228 (59.53)	92 (57.86)	76 (58.91)	47 (65.28)	13 (56.52)	.734
Antihyperlipidemic drugs, n (%)	273 (71.28)	111 (69.81)	95 (73.64)	53 (73.61)	14 (60.87)	.587
Anticoagulant drugs, n (%)	27 (7.05)	10 (6.29)	8 (6.20)	8 (11.11)	1 (4.35)	.503
Antihypertensive drugs, n (%)	298 (77.81)	127 (79.87)	101 (78.29)	57 (79.17)	13 (56.52)	.089
Antihyperglycemic drugs, n (%)	88 (22.98)	42 (26.42)	30 (23.26)	14 (19.44)	2 (8.70)	.238
Laboratory indicators						
Blood glucose on admission (mmol/L)	6.52 (5.63–8.25)	6.38 (5.61–8.03)	6.54 (5.69–8.50)	6.54 (5.79–8.55)	6.85 (5.51–8.16)	.725
Total cholesterol (mmol/L)	4.57 (3.88–5.29)	4.68 (4.05–5.40)	4.52 (3.96–5.28)	4.47 (3.65–5.17)	4.37 (3.65–5.24)	.217
Triglycerides (mmol/L)	1.58 (1.12–2.28)	1.68 (1.18–2.52)	1.50 (1.10–1.99)	1.57 (1.12–2.40)	1.36 (0.96–1.89)	.075
High-density lipoprotein cholesterol (mmol/L)	1.16 (1.00–1.38)	1.15 (0.99–1.34)	1.21 (1.04–1.42)	1.12 (1.00–1.33)	1.16 (0.94–1.33)	.160
Low-density lipoprotein cholesterol (mmol/L)	2.85 (2.25–3.45)	2.98 (2.38–3.58)	2.78 (2.29–3.35)	2.77 (2.10–3.27)	2.79 (2.00–3.43)	.204
Lipoprotein a (mg/L)	144.00 (66.00–315.50)	134.00 (64.00–324.00)	134.00 (59.00–254.00)	147.50 (82.25–346.25)	265.00 (92.00–392.50)	.331
Arteriosclerosis index	0.51 (0.41–0.65)	0.47 (0.38–0.60)	0.54 (0.43–0.66)	0.60 (0.44–0.72)	0.56 (0.46–0.62)	<.001*
Blood pressure monitoring indicators						
24 h-SBP (mm Hg)	134.00 (123.00–150.00)	133.00 (122.00–153.00)	133.00 (125.00–146.00)	138.50 (123.50–152.00)	141.00 (126.50–157.00)	.369
24 h-DBP (mm Hg)	73.00 (65.00–81.00)	76.00 (68.00–84.00)	72.00 (64.00–80.00)	69.50 (61.00–78.00)	70.00 (66.50–79.00)	.005***
SD of 24 h-SBP (mm Hg)	16.65 (13.61–19.27)	16.69 (13.50–19.20)	16.05 (14.10–19.10)	16.93 (12.97–19.93)	16.80 (14.61–20.30)	.845
SD of 24 h-DBP (mm Hg)	10.57 (8.70–13.60)	11.37 (9.53–13.87)	10.12 (8.66–13.16)	9.59 (7.63–13.25)	10.71 (7.56–13.70)	.005***
Daytime SBP (mm Hg)	135.00 (124.50–151.00)	135.00 (125.00–153.50)	134.00 (125.00–148.00)	137.50 (122.00–150.25)	136.00 (123.50–157.50)	.582
Daytime DBP (mm Hg)	74.00 (66.00–82.00)	77.00 (69.00–87.00)	71.00 (64.00–81.00)	69.50 (62.75–78.25)	72.00 (67.00–77.50)	<.001*
Nighttime SBP (mm Hg)	135.00 (120.00–150.00)	129.00 (114.00–146.00)	135.00 (123.00–150.00)	137.00 (121.75–153.00)	147.00 (131.00–158.50)	.006***
Nighttime DBP (mm Hg)	71.00 (63.00–79.00)	72.00 (64.00–80.00)	71.00 (64.00–78.00)	69.50 (61.00–77.00)	71.00 (65.50–75.50)	.404
SBP nocturnal decline rate	1.70 (−3.70–7.55)	5.60 (0.00–11.00)	0.00 (−4.30–4.80)	0.00 (−5.25–4.05)	−3.40 (−9.10–0.90)	<.001*
DBP nocturnal decline rate	3.30 (−0.60–9.10)	6.80 (2.00–12.15)	1.60 (−1.70–6.90)	0.70 (−1.75–5.92)	0.00 (−5.65–2.30)	<.001*

DBP = diastolic blood pressure, MMSE = minimum mental state examination, SBP = systolic blood pressure, SD = standard deviation.

* *P* <.001.

** *P* <.05.

*** *P* <.01.

### 3.2. Relationship between diastolic blood pressure variability, arteriosclerosis index and different degrees of cognitive dysfunction

**Figure 1.**
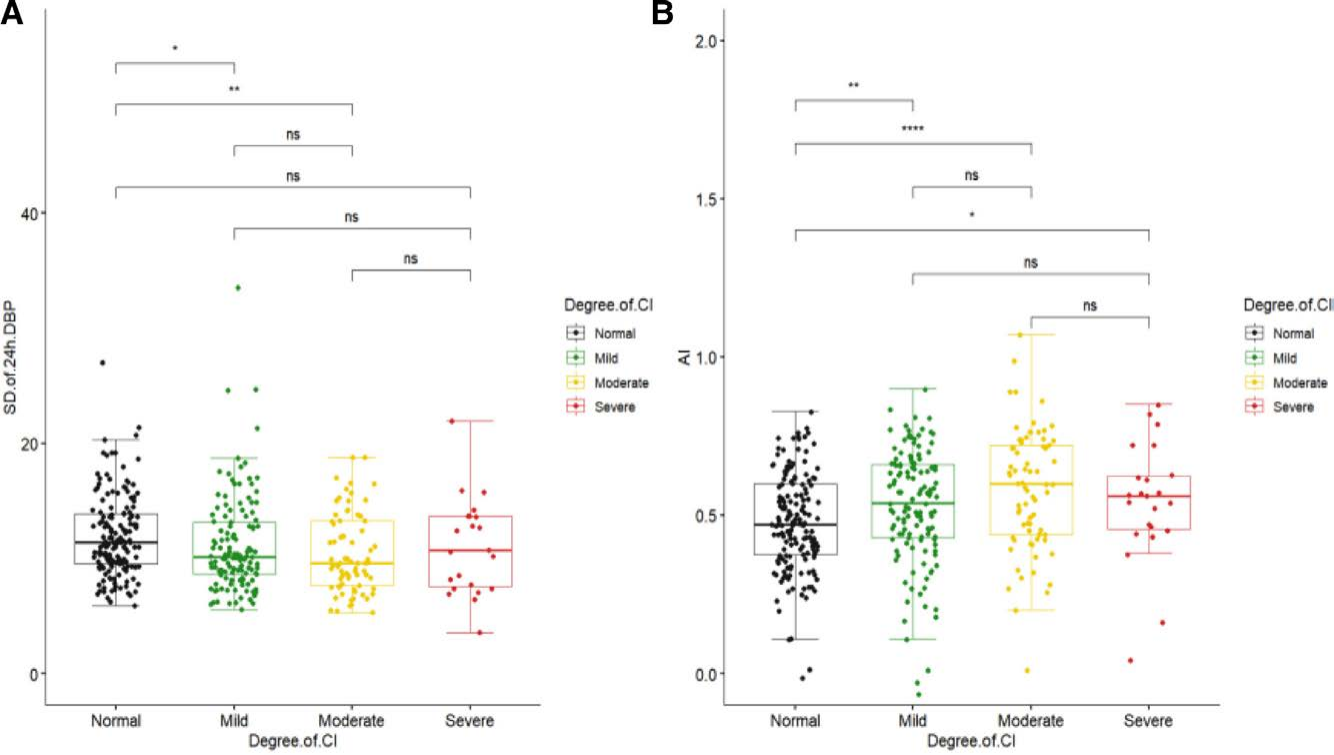
Relationship between 24-h mean diastolic blood pressure standard deviation, arteriosclerosis index, and degree of cognitive impairment. (A) Differences in 24-h mean diastolic blood pressure standard deviation across cognitive function groups. (B) Differences in arteriosclerosis index across cognitive function groups. AI = arteriosclerosis index, Degree of CI = degree of cognitive impairment, ns = no significance, SD of 24h-DBP = standard deviation of 24-hour diastolic blood pressure. *P <.05, **P <.01, ***P <.001, ****P <.0001.

**Figure 2.**
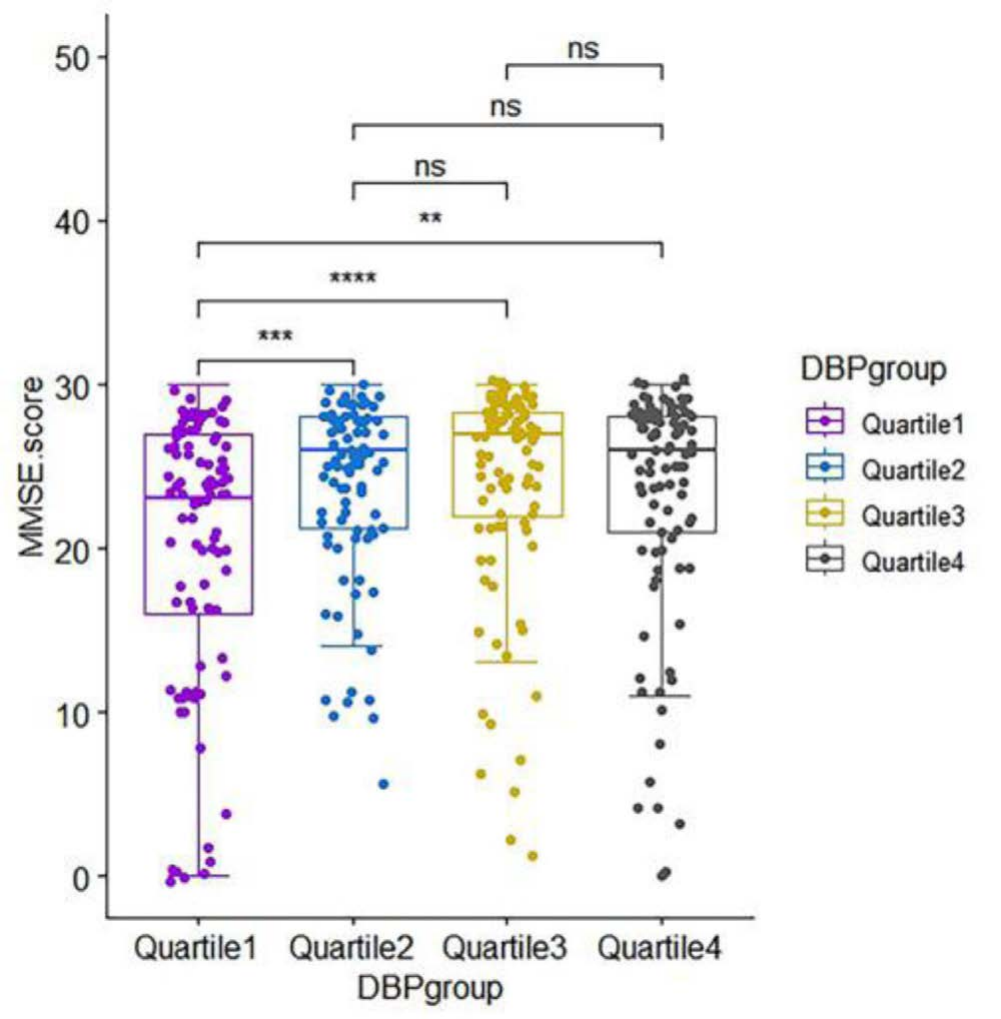
Differences in standard deviation of 24-h mean diastolic blood pressure by quartile intergroup MMSE scores intergroup MMSE scores. DBP group = diastolic blood pressure group, MMSE = minimum mental state examination, ns = no significance. *P <.05, **P <.01, ***P <.001, ****P <.0001.

Figure 1 shows the relationship between the SD of 24h-DBP, AI, and the degree of cognitive dysfunction. In Figure 1A, we observed that the SD of 24h-DBP in the mild and moderate cognitive dysfunction groups was lower than that in the normal cognitive function group. The AI was higher in the mild and moderate cognitive dysfunction group than in the normal cognitive function group in Figure 1B. We performed between-group comparisons of SD of 24h-DBP by quartile grouping, as shown in Table 2. Among the included participants, Quartile 1 was relatively older and had the highest number of people with cognitive dysfunction (73.47%), while the MMSE score was relatively low. AI was higher in Quartile 1 than in the other 3 groups. Blood pressure monitoring indices of the 4 groups included 24h-SBP, 24h-DBP, SD of 24h-SBP, SD of 24h-DBP, dmSBP, dmDBP, nmSBP, nmDBP, nocturnal SBP drop rate and nocturnal DBP drop rate with intergroup differences (*P *< .05). Afterwards, we performed a comparison of MMSE scores among the 4 groups, and in Figure 2, MMSE scores of Quartile 2, Quartile 3, and Quartile 4 were higher than those of Quartile 1 (*P* < .05), and there was no difference among the groups of Quartile 2, Quartile 3, and Quartile 4.

**Table 2:** Baseline characteristics according to standard deviation of 24-h diastolic blood pressure by quartile.

Characteristics	Total (n = 383)	Quartile 1 (n = 98)	Quartile 2 (n = 94)	Quartile 3 (n = 96)	Quartile 4 (n = 95)	*P*-value
Age (years)	74.00 (65.00–83.00)	80.00 (70.00–87.00)	73.00 (66.00–82.00)	70.00 (64.00–82.00)	74.00 (64.00–83.00)	.001*
Gender, male, n (%)	171 (44.65)	42 (42.86)	35 (37.23)	48 (50.00)	46 (48.42)	.275
Smoking, n (%)	68 (17.75)	15 (15.31)	14 (14.89)	16 (16.67)	23 (24.21)	.293
Drinking, n (%)	68 (17.75)	15 (15.31)	14 (14.89)	16 (16.67)	23 (24.21)	.631
MMSE score	25.00 (21.00–28.00)	23.00 (16.00–27.00)	26.00 (21.25–28.00)	27.00 (22.00–28.25)	26.00 (21.00–28.00)	<.001**
Cognitive dysfunction, n (%)	224 (58.49)	72 (73.47)	55 (58.51)	44 (45.83)	53 (55.79)	.001*
Degree of cognitive dysfunction						
Severe cognitive impairment, n (%)	23 (6.01)	9 (9.18)	1 (1.06)	6 (6.25)	7 (7.37)	.003*
Moderate cognitive impairment, n (%)	72 (18.8)	28 (28.57)	16 (17.02)	12 (12.50)	16 (16.84)	
Mild cognitive impairment, n (%)	129 (33.68)	35 (35.71)	38 (40.43)	26 (27.08)	30 (31.58)	
Normal cognition, n (%)	159 (41.51)	26 (26.53)	39 (41.49)	52 (54.17)	42 (44.21)	
Past medical history						
Hypertension, n (%)	306 (79.9)	74 (75.51)	80 (85.11)	77 (80.21)	75 (78.95)	.420
Diabetes, n (%)	92 (24.02)	24 (24.49)	26 (27.66)	19 (19.79)	23 (24.21)	.651
Stroke or TIA, n (%)	137 (35.77)	41 (41.84)	31 (32.98)	34 (35.42)	31 (32.63)	.512
Cardiological history, n (%)	130 (33.94)	45 (45.92)	33 (35.11)	25 (26.04)	27 (28.42)	.016***
Drug treatment history						
Antiplatelet drugs, n (%)	228 (59.53)	64 (65.31)	60 (63.83)	41 (42.71)	63 (66.32)	.002*
Antihyperlipidemic drugs, n (%)	273 (71.28)	70 (71.43)	73 (77.66)	58 (60.42)	72 (75.79)	.039***
Anticoagulant drugs, n (%)	27 (7.05)	8 (8.16)	4 (4.26)	3 (3.12)	12 (12.63)	.044***
Antihypertensive drugs, n (%)	298 (77.81)	75 (76.53)	80 (85.11)	73 (76.04)	70 (73.68)	.251
Antihyperglycemic drugs, n (%)	88 (22.98)	17 (17.35)	27 (28.72)	22 (22.92)	22 (23.16)	.319
Laboratory indicators						
Blood glucose on admission (mmol/L)	6.52 (5.63–8.25)	6.49 (5.61–8.13)	6.54 (5.75–9.08)	6.75 (5.63–8.11)	6.33 (5.62–8.12)	.743
Total cholesterol (mmol/L)	4.57 (3.88–5.29)	4.42 (3.65–5.06)	4.58 (3.90–5.51)	4.74 (4.17–5.31)	4.51 (4.05–5.43)	.216
Triglycerides (mmol/L)	1.58 (1.12–2.28)	1.56 (1.09–2.41)	1.49 (1.09–2.08)	1.51 (1.09–2.13)	1.75 (1.27–2.58)	.120
High-density lipoprotein cholesterol (mmol/L)	1.16 (1.00–1.38)	1.12 (0.97–1.33)	1.14 (0.96–1.43)	1.23 (1.03–1.39)	1.18 (1.04–1.38)	.435
Low-density lipoprotein cholesterol (mmol/L)	2.85 (2.25–3.45)	2.75 (2.10–3.19)	2.78 (2.23–3.50)	3.15 (2.42–3.46)	2.84 (2.29–3.52)	.057
Lipoprotein a (mg/L)	144.00 (66.00–315.50)	139.50 (63.00–320.50)	160.50 (68.50–329.25)	127.50 (67.25–294.75)	138.00 (71.00–300.00)	.942
Arteriosclerosis index	0.51 (0.41–0.65)	0.66 (0.57–0.74)	0.56 (0.47–0.65)	0.50 (0.41–0.56)	0.40 (0.27–0.47)	<.001**
Blood pressure monitoring indicators						
24 h-SBP (mm Hg)	134.00 (123.00–150.00)	129.00 (116.50–142.00)	130.50 (124.00–148.00)	137.00 (124.75–153.25)	141.00 (130.00–159.00)	<.001**
24 h-DBP (mm Hg)	73.00 (65.00–81.00)	67.00 (61.00–75.00)	71.00 (64.00–78.00)	76.50 (70.00–84.00)	80.00 (68.50–86.50)	<.001**
SD of 24 h-SBP (mm Hg)	16.65 (13.61–19.27)	13.35 (11.10–15.48)	15.20 (13.53–17.82)	17.60 (15.30–19.25)	19.94 (17.70–22.30)	<.001**
SD of 24 h-DBP (mm Hg)	10.57 (8.70–13.60)	7.40 (6.81–8.10)	9.62 (9.24–10.06)	11.89 (11.19–12.80)	15.80 (14.43–17.16)	<.001**
Daytime SBP (mm Hg)	135.00 (124.50–151.00)	130.00 (119.00–141.75)	133.00 (123.25–149.00)	140.50 (126.00–153.00)	141.00 (129.00–161.00)	<.001**
Daytime DBP (mm Hg)	74.00 (66.00–82.00)	66.00 (61.25–74.75)	71.00 (65.50–79.00)	77.50 (71.00–84.00)	81.00 (70.00–88.00)	<.001**
Nighttime SBP (mm Hg)	135.00 (120.00–150.00)	128.50 (115.25–149.00)	130.00 (118.50–143.00)	138.00 (121.25–152.25)	139.00 (123.00–158.00)	.011***
Nighttime DBP (mm Hg)	71.00 (63.00–79.00)	66.00 (59.00–74.00)	69.00 (61.00–75.00)	73.00 (65.00–83.00)	74.00 (66.00–82.00)	<.001**
SBP nocturnal decline rate	1.70 (−3.70 to 7.55)	0.00 (−4.90 to 4.15)	2.65 (−3.00 to 6.87)	1.85 (−5.12 to 11.22)	2.40 (−1.40 to 8.70)	.013***
DBP nocturnal decline rate	3.30 (−0.60 to 9.10)	0.00 (−2.82 to 3.88)	4.00 (0.00–7.58)	4.05 (−1.12 to 11.08)	6.30 (0.05–12.55)	<.001**

DBP = diastolic blood pressure, MMSE = minimum mental state examination, SBP = systolic blood pressure, SD = standard deviation.

* *P* <.01.

** *P* <.001.

*** *P* <.05.

### 3.3. Relationship between blood pressure variability, arteriosclerosis index, and MMSE scores

**Figure 3.**
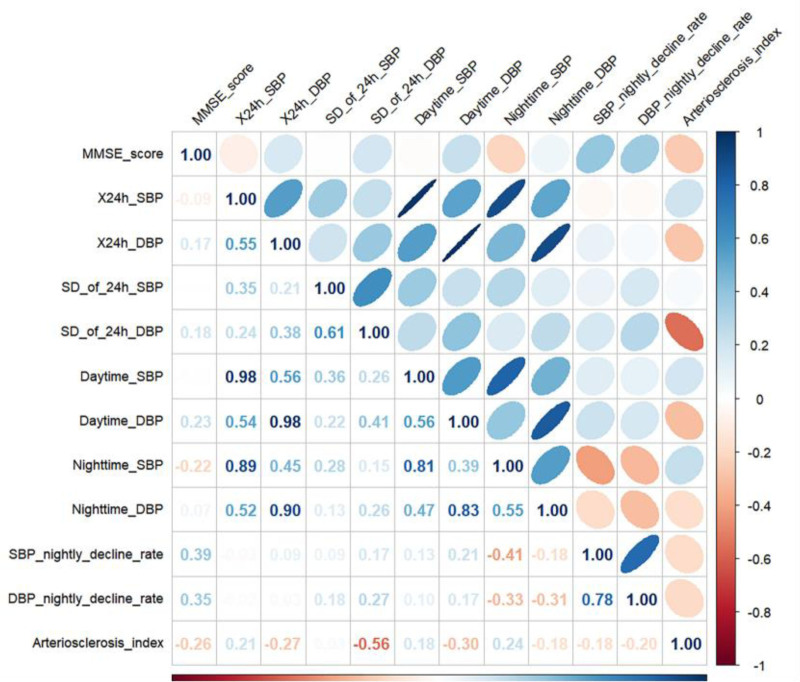
Heatmap of correlation between MMSE scores, blood pressure monitoring indices and arterioscleros indices. Red indicates a negative correlation, blue indicates a positive correlation, and the darker the color, the greater the correlation. The roundness of the graph also represents correlation; the rounder the graph, the greater the correlation. DBP = diastolic blood pressure, MMSE = minimum mental state examination, SBP = systolic blood pressure, SD = standard deviation.

**Figure 4.**
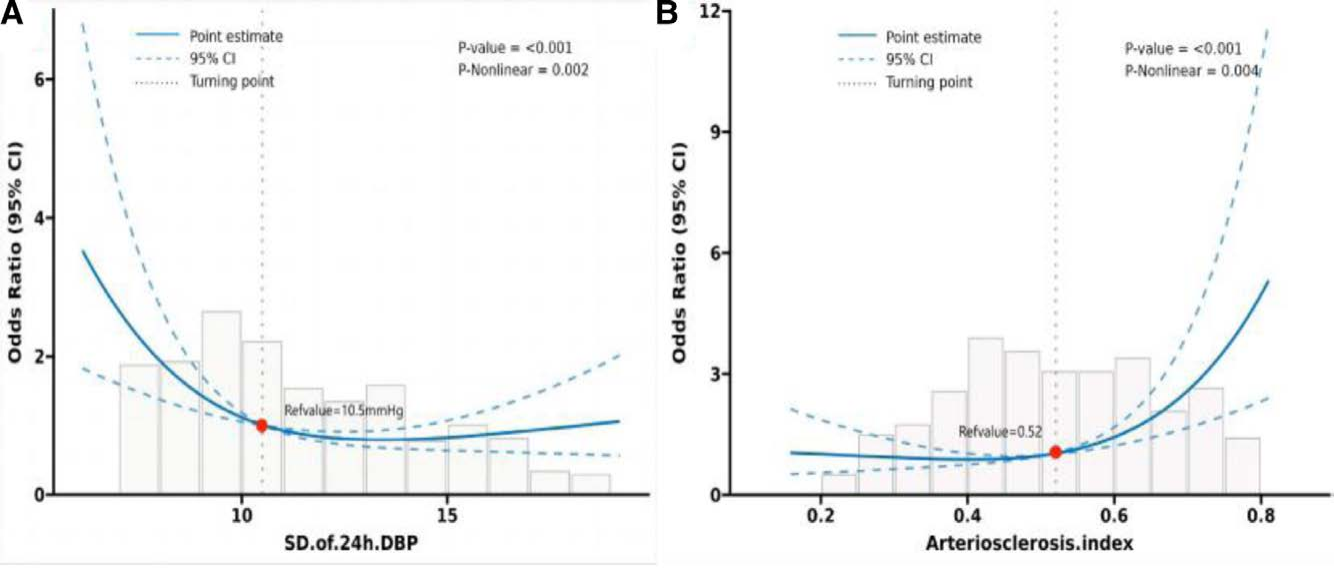
Restricted cubic spline curves for standard deviation of 24-hour diastolic blood pressure, arteriosclerosis index, and MMSE score. CI = confidence interval, OR = odds ratio, SD of 24h-DBP = standard deviation of 24-hour diastolic blood pressure.

Figure 3 shows the correlation heatmap of MMSE score, BPV and AI, in which SD of 24h-DBP was negatively correlated with the AI (*r* = −0.56, *P* < .001), whereas it was positively correlated with MMSE score (*R* = 0.18, *P* = .024). AI was negatively correlated with MMSE score (*r* = −0.26, *P* < .001). Table 3 demonstrates the relationship between blood pressure monitoring indices, AI and MMSE score, in which there was no significant relationship between MMSE score and 24h-SBP, 24h-DBP, SD of 24h-SBP, dmSBP, and nmDBP. The MMSE score had a negative correlation with the 24h-DBP (*R* = 0.181, *P* = .024), dmDBP (*R* = 0.228, *P* < .001), nocturnal SBP decrease rate (*R* = 0.387, *P* < .001), and nocturnal DBP decrease rate (*R* = 0.355, *P* < .001) were significantly and positively correlated. MMSE score was significantly and positively correlated with nmSBP (*r* = −0.219, *P* = .001), AI (*r* = −0.257, *P* < .001) were significantly negatively correlated. RCS is a common method to explore whether there is a nonlinear association between independent and dependent variables.^[20]^ From the dose-relationship plot (Fig. 4), a nonlinear relationship was found between SD of 24h-DBP, AI and MMSE score (overall *P* < .05, nonlinear *P* < .05), and when the SD of 24h-DBP was <10.5 mm Hg, the lower the MMSE score was as the SD of 24h-DBP increased. When the AI was >0.52, as the AI increased the MMSE score was higher.

**Table 3:** Relationship between blood pressure monitoring indicators, arterioscleros index and MMSE scores.

	MMSE score	
Parameter	*r*	*P*
24 h-SBP (mm Hg)	−0.086	>.999
24 h-DBP (mm Hg)	0.170	.056
SD of 24 h-SBP (mm Hg)	−0.006	>.999
SD of 24 h-DBP (mm Hg)	0.181	.024*
Daytime SBP (mm Hg)	−0.018	>.999
Daytime DBP (mm Hg)	0.228	<.001***
Nighttime SBP (mm Hg)	−0.219	.001**
Nighttime DBP (mm Hg)	0.068	>.999
SBP nocturnal decline rate	0.387	<.001***
DBP nocturnal decline rate	0.355	<.001***
Arteriosclerosis index	−0.257	<.001***

DBP = diastolic blood pressure, MMSE = minimum mental state examination, SBP = systolic blood pressure, SD = standard deviation.

* *P* <.05.

** *P* <.01.

*** *P* <.001.

### 3.4. Binary logistic regression to explore the relationship between diastolic blood pressure, arteriosclerosis index and cognitive dysfunction

**Figure 5.**
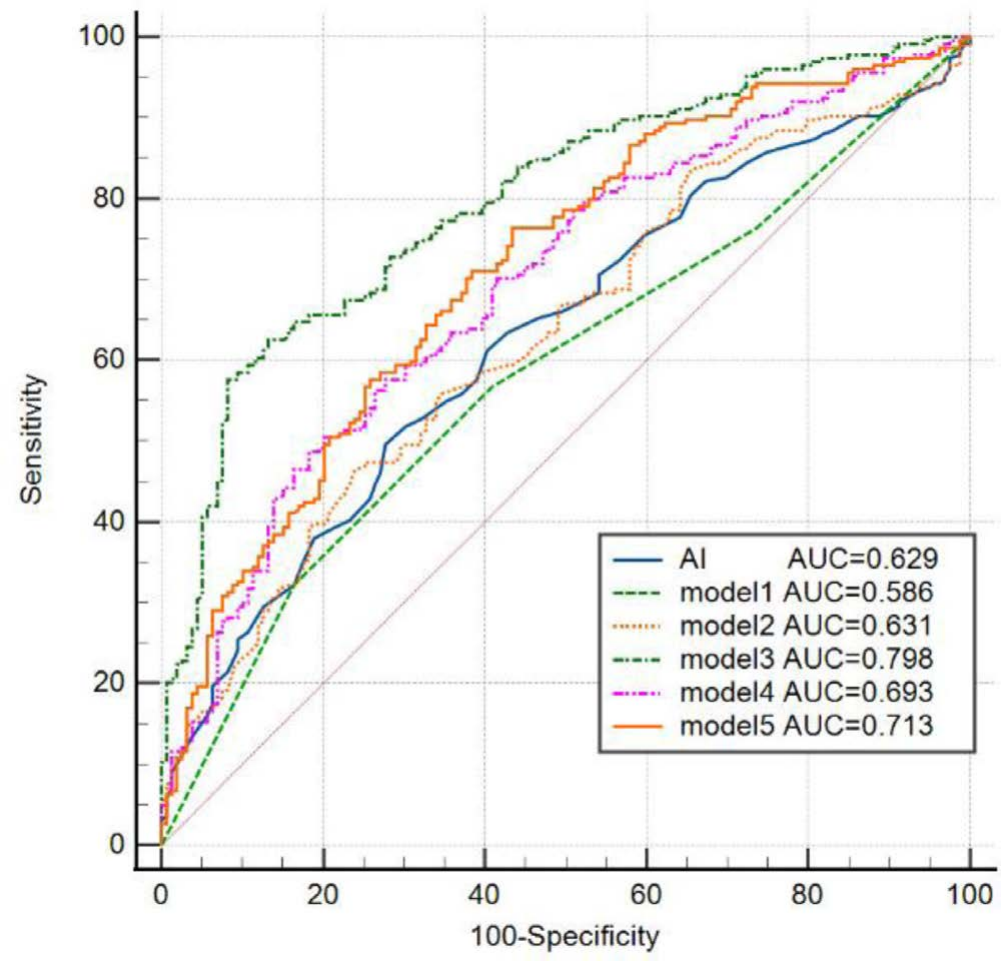
Receiver operating characteristic curves for the 5 models. AI = arteriosclerosis index, AUC = area under curve.

Table 4 demonstrates the multivariate logistic regression analyses between SD of 24h-DBP and AI and vascular cognitive dysfunction in different models. Model 1 is a univariate analysis of the SD of 24-hour DBP (24h-DBP) and cognitive dysfunction. Compared with the Quartile 1 group, the risk of cognitive dysfunction was lower in the Quartile 2, Quartile 3, and Quartile 4 groups. Specifically, participants in Quartile 2 had a lower risk of cognitive dysfunction (Model 1: odds ratio (OR) = 0.51, 95% confidence interval (CI) = 0.28–0.94), participants in Quartile 3 had a lower risk as well (OR = 0.31, 95% CI = 0.17–0.56), and those in Quartile 4 also had a lower risk (OR = 0.46, 95% CI = 0.25–0.83), with a *P*-value < 0.001. Model 2 is the adjusted AI model, from which the AI was seen as a risk factor for vascular cognitive dysfunction (OR = 8.00, 95CI%=1.89–33.77; *P* < .01). The risk of cognitive dysfunction was significantly lower in the Quartile 3 group in all models. The SD of 24h-DBP and AI were independent of all covariates after adjusting for age, sex, smoking, alcohol consumption, history of hypertension, diabetes mellitus, cerebrovascular disease, cardiac disease, and use of antiplatelet medications, antihypertensive drugs, anticoagulants, lipid-lowering drugs, and hypoglycemic drugs. We assessed the 5 models by the ROC curve, with the model 3 curve having the largest area under the curve of 0.798 (Fig. 5).

**Table 4:** Multivariate logistic regression analysis between standard deviation of 24-hour diastolic blood pressure and arterioscleros index and vascular cognitive dysfunction in different models.

Model	Quartile 1	Quartile 2	Quartile 3	Quartile 4	Arteriosclerosis index	*P*-value
1	Reference	0.51 (0.28–0.94)*	0.31 (0.17–0.56)***	0.46 (0.25–0.83)*	NA	<.001***
2	Reference	0.58 (0.31–1.08)	0.41 (0.22–0.77)**	0.77 (0.38–1.55)	8.00 (1.89–33.77)**	.005**
3	Reference	0.64 (0.32–1.30)	0.43 (0.21–0.89)*	0.61 (0.27–1.36)	0.97 (0.18–5.31)	.022*
4	Reference	0.64 (0.33–1.25)	0.43 (0.22–0.86)*	0.88 (0.41–1.87)	8.55 (1.81–40.50)**	.016*
5	Reference	0.62 (0.31–1.22)	0.44 (0.22–0.89))*	0.91 (0.42–1.99)	8.66 (1.75–42.91)**	.022*

TIA = transient ischemic attack.

Model 1: crude model; model 2: adjusted for arteriosclerosis index; model 3: adjusted for arteriosclerosis index,age and sex; model 4: adjusted for arteriosclerosis index,age, sex, smoking, drinking, Hypertension, diabetes, stroke/TIA, cardiology history, use of antiplatelet medications, antihypertensive drugs, anticoagulants, lipid-lowering drugs, and hypoglycemic drugs; model 5: adjusted for adjusted for arteriosclerosis index,age, sex, smoking, drinking, hypertension, diabetes, stroke/ TIA, cardiology history, use of antiplatelet medications, antihypertensive drugs, anticoagulants, lipid-lowering drugs, hypoglycemic drugs, total cholesterol, triglycerides, high-density lipoprotein, low-density lipoprotein, lipoprotein a and blood glucose on admission.

* *P* <.05.

** *P* <.01.

*** *P* <.001.

## 4. Disscussion

This study reveals there were differences between groups in 24h-DBP, SD of 24h-DBP, dmDBP, nmSBP, and nocturnal rates of decrease in systolic and DBP in patients with different degrees of cognitive dysfunction. Notably, the variability in 24h-DBP is significantly lower in the mild and moderate cognitive dysfunction groups compared to the normal cognitive function group. Additionally, the arterial stiffness index negatively correlates with cognitive decline, while showing a positive correlation with the 24-hour average DBPV. In other words, Individuals with lower SD of 24h-DBP exhibited higher AI and poorer cognitive functions, emphasizing the importance of diastolic pressure stability in maintaining cognitive health. Unlike previous studies that mainly focused on the impact of systolic pressure on cognitive function, our findings suggest that variability in diastolic pressure may be an overlooked risk factor for cognitive decline.

### 4.1. Blood pressure and cerebral small blood vessel disease

The relationship between blood pressure and cognitive impairment in CSVD is complex and multidimensional. Mean premorbid SBP (the average SBP recorded before the development of CSVD) is more strongly associated with CSVD burden than baseline SBP. The relationship between baseline diastolic BP and development of CSVD is complex. Mean DBP at baseline was negatively associated with CSVD score and a positive association was only evident for DBP 10 to 20 years previously.^[21]^ Another study is focused on the pathophysiology of Alzheimer disease through the linkage between sleep apnea and abnormal circadian blood pressure rhythm.^[22]^ Yuan study points out that increased 24h-DBP, nmSBP and nmDBP and SD of 24-hour DBP were independently correlated with CSVD occurrence, suggesting that sympathetic overactivity plays a role in the pathogenesis of CSVD.^[23]^ Ide et al reported that increased SBP, DBP, and mean BP were associated with a risk of the progression of CSVD examined by magnetic resonance imaging in healthy cohort population.^[24]^ One study included participants with CSVD from the Radboud University Nijmegen Diffusion Tensor Magnetic resonance-imaging Cohort. There was no association between systolic BPV and gray and white matter volumes, Peak skeleton of mean diffusivity or microbleed count after 13.7 years. BPV is associated with increased progression of white matter hyperintensity volumes and higher risk of incident lacunes over 14 years in participants with CSVD.^[25]^ Another study by Sveikata L show Long-term SBPV has a dose-dependent association with alterations in white matter integrity, lobar lacunes, and cortical cerebral microinfarcts, and predicts cognitive decline. They emphasized the role of BPV in contributing to vessel wall damage and cognitive dysfunction.^[26]^ Our study is slightly different from the above studies in that it attempts to explore the effects of blood pressure on CSVD at different cognitive levels. Our comparison of 24-hour blood pressure monitoring indices in patients with varying degrees of cognitive dysfunction revealed significant differences in nocturnal SBP as well as in the 24-h SBP between the groups.

### 4.2. Arteriosclerosis index and cognitive decline

AI has been linked to heightened risks for cognitive decline, and ultimately for dementia.^[27–29]^ Bowie study suggests that, through diffuse optical imaging of the cerebral arterial pulse, there is a sharp decline in cerebral arterial elasticity and an acceleration in the development of white matter lesions by nearly a decade in women after menopause.^[30]^ This suggests that age may impair cognition via the sequential indirect effects of arteriosclerosis and white matter atrophy on fluid, but not crystallized, abilities. A total of 1554 healthy middle-aged and older adults participated a study of vascular stiffness as measured by brachial-ankle pulse wave velocity suggesting that increased arterial stiffness is associated with impaired cognitive function in middle-aged and older adults.^[31]^ A total of 127 cognitively impaired individuals participated in a study that used the Ankle-Brachial Index as an indicator of AI. The findings suggest that lower ABI levels contribute to the aggravation of dementia-related regional neurodegeneration in older adults with cognitive impairment.^[32]^ The mechanisms by which arterial stiffness is associated with cognitive decline are unknown; possible mechanisms include inflammation, nitrooxidative stress, impaired autophagy, and insulin resistance.^[27]^ In our study, we chose the AI, which consists of TC and HDL, to assess the degree of arterial stiffness. Our study found that AI was negatively correlated with cognitive decline. This finding is generally consistent with these studies above.

### 4.3. Diastolic blood pressure and cerebrovascular disease

DBP is the pressure of blood on the artery walls during the heart’s relaxation phase and represents the lower value in blood pressure readings. The level of DBP significantly affects cerebral blood flow, thereby having a direct impact on the risk and progression of cerebrovascular diseases.^[33]^ A study examines the effects of blood pressure variations on cerebrovascular health, utilizing long-term data from the Arteriosclerosis Risk in Communities study. It emphasizes the role of blood pressure, particularly DBP, in elevating the risk of cerebrovascular diseases and offers insights into the mechanisms through which high DBP may lead to an increased risk of cerebral arteriosclerosis.^[34]^ In addition, high DBP is associated with a variety of cerebrovascular diseases, including stroke, cerebral hemangioma, and vascular cognitive dysfunction.^[35]^ On the other hand, excessively low DBP may reduce blood flow to the brain, resulting in an inadequate supply of oxygen and nutrients to brain tissue. Chronic low DBP may increase the risk of cerebral ischemia, which increases the risk of mild cognitive impairment to serious cerebrovascular accidents.^[36]^ Significant fluctuations in DBP, especially frequent high and low changes, are also considered a risk factor for cerebrovascular disease.^[37]^ Our research has found that individuals with lower SD of 24-hour DBP exhibited higher AI and poorer cognitive functions, emphasizing the importance of DBP stability in maintaining cognitive health. Unlike previous studies that mainly focused on the impact of systolic pressure on cognitive function, our findings suggest that variability in DBP may be an overlooked risk factor for cognitive decline.

### 4.4. Diastolic blood pressure variability and arteriosclerosis index

There is an intrinsic link between DBPV and the AI, which is reflected in the fact that both are closely related to functional and structural changes in blood vessels. DBPV usually refers to the degree to which an individual’s DBP fluctuates at different points in time, whereas the AI, which is usually indexed by the thickness of the blood vessel wall or the elasticity of the blood vessel, reflects the degree of hardening of the blood vessel. One study by YG Tedla shows that vascular changes can stiffen the arterial wall, impacting the elasticity modulus of arterial wall across different SBP.^[38]^ Increased DBPV may cause the vessel wall to undergo changing pressures, which in turn increases vascular endothelial cell damage and inflammation, factors that are early drivers in the development of arteriosclerosis.^[39]^ The study of M Amir demonstrates how variations in blood flow, especially under conditions like stenosis, can influence vascular wall pressure and blood flow characteristics. Through computational fluid dynamics, illustrates the dynamic interactions between blood flow patterns and vascular health, revealing how altered flow characteristics can affect endothelial function and contribute to vascular remodeling. These interactions may lead to changes in wall shear stress, pressure gradients, and flow turbulence. This research underscores the potential of unstable DBP to cause similar hemodynamic perturbations, affecting the vascular wall’s pressure sensing mechanisms and altering blood flow characteristics in a manner that may expedite the process of arteriosclerosis.^[40]^ The progression of arteriosclerosis leads to increased vessel wall thickness and decreased vessel elasticity. This alteration can further exacerbate DBP fluctuations, as hardened blood vessels are unable to effectively regulate blood pressure changes, resulting in increased DBPV.^[41]^ Yet there is also a phenomenon of vascular failure, in which stiff vessels lead to worse DBPV and diminished or even no significant change in DBPV.^[26]^ This observation may explain the finding of our study that the older the vessel, the stiffer the vessel, and the worse the DBPV.

However, this study has several limitations: first, as an observational study, this study only identified correlations but not causation. Second, the lack of longitudinal data and potential confounders (e.g., lifestyle or genetics) leaves a gap in understanding the development of cognitive impairment and the factors that influence it. In addition to this, the study lacks insight into mechanisms and intervention testing that could elucidate the effects of altered blood pressure changes or arterial stiffness on cognitive outcomes. Future research addressing these issues could enhance intervention strategies for CSVD induced cognitive impairment.

In conclusion, this study reveals a significant link between DBPV, arterial stiffness, and cognitive impairment in CSVD patients. It shows that lower DBPV and higher arterial stiffness independently correlate with cognitive dysfunction. These findings underscore the importance of vascular health in managing CSVD and preventing cognitive decline, suggesting that controlling BPV and arterial stiffness could be crucial.

## Acknowledgments

The authors are grateful to Dr Xiaozhu Shen for financial support. The authors thank all the nurses in the Department of Geriatrics of the Second People’s Hospital of Lianyungang City, Jiangsu Province, China, for their support in collecting patient information for our tests.

## Author contributions

**Conceptualization:** Wen Zhong, Lingling Wang.

**Data curation:** Lingling Wang, Yiwen Xu.

**Formal analysis:** Wen Zhong, Yiwen Xu.

**Funding acquisition:** Xiaozhu Shen.

**Supervision:** Likai Jin.

**Writing – original draft:** Wen Zhong, Mengqian Liu, Yi Jiang.

**Writing – review & editing:** Likai Jin, Xiaozhu Shen.
